# dNTP pool modulation dynamics by SAMHD1 protein in monocyte-derived macrophages

**DOI:** 10.1186/s12977-014-0063-2

**Published:** 2014-08-27

**Authors:** Joseph A Hollenbaugh, Sijia Tao, Gina M Lenzi, Sulryung Ryu, Dong-Hyun Kim, Felipe Diaz-Griffero, Raymond F Schinazi, Baek Kim

**Affiliations:** Department of Pediatrics, Center for Drug Discovery, Emory Center for AIDS Research, Laboratory of Biochemical Pharmacology, Emory University School of Medicine, 1760 Haygood Drive, Health Sciences Research Building, Atlanta, Georgia 30322 USA; Department of Microbiology and Immunology, Albert Einstein College of Medicine Bronx, Bronx, NY 10461 USA; Veterans Affairs Medical Center, Decatur, Georgia 30033 USA; College of Pharmacy, Kyung-Hee University, Seoul, South Korea

**Keywords:** HIV-1, Monocytes-derived macrophages, Vpx, SAMHD1, dNTPs: virus-like particles

## Abstract

**Background:**

SAMHD1 degrades deoxyribonucleotides (dNTPs), suppressing viral DNA synthesis in macrophages. Recently, viral protein X (Vpx) of HIV-2/SIVsm was shown to target SAMHD1 for proteosomal degradation and led to elevation of dNTP levels, which in turn accelerated proviral DNA synthesis of lentiviruses in macrophages.

**Results:**

We investigated both time-dependent and quantitative interplays between SAMHD1 level and dNTP concentrations during multiple exposures of Vpx in macrophages. The following were observed. First, SAMHD1 level was rapidly reduced by Vpx + VLP to undetectable levels by Western blot analysis. Recovery of SAMHD1 was very slow with less than 3% of the normal macrophage level detected at day 6 post Vpx treatment and only ~30% recovered at day 14. Second, dGTP, dCTP and dTTP levels peaked at day 1 post Vpx treatment, whereas dATP peaked at day 2. However, all dNTPs rapidly decreased starting at day 3, while SAMHD1 level was below the level of detection. Third, when Vpx pretreated macrophages were re-exposed to a second Vpx treatment at day 7, we observed dNTP elevation that had faster kinetics than the first Vpx + VLP treatment. Moreover, we performed a short kinetic analysis of the second Vpx treatment to find that dATP and dGTP levels peaked at 8 hours post secondary VLP treatment. dGTP peak was consistently higher than the primary, whereas peak dATP concentration was basically equivalent to the first Vpx + VLP treatment. Lastly, HIV-1 replication kinetics were faster in macrophages treated after the secondary Vpx treatments when compared to the initial single Vpx treatment.

**Conclusion:**

This study reveals that a very low level of SAMHD1 sufficiently modulates the normally low dNTP levels in macrophages and proposes potential diverse mechanisms of Vpx-mediated dNTP regulation in macrophages.

**Electronic supplementary material:**

The online version of this article (doi:10.1186/s12977-014-0063-2) contains supplementary material, which is available to authorized users.

## Background

Sterile alpha motif (SAM) and histidine/aspartic acid (HD) domain protein 1 (SAMHD1) has been linked to Aicardi-Goutières Syndrome, which is a rare autoimmune disease [1]. In addition to its role in autoimmunity [2-4], SAMHD1 has been studied in the context of antiviral response [1,5-12] and genomic stability [2,13]. Several groups have now shown that SAMHD1 is found in all cell types and localizes to the nucleus [5,10,14-19].

Recent evidence indicates that SAMHD1 has at least two different cellular functions. First, SAMHD1 was shown to have deoxyribonucleoside triphosphate (dNTP) phosphohydrolase activity [20,21], suggesting it is a host antiviral restriction factor to limit replication of retroviral and DNA containing viruses by depleting cellular dNTPs in viral non-dividing target cell types [22-25]. Recently, both biochemical and structural evidence indicated that SAMHD1 forms a tetramer as the active dNTP phosphohydrolase complex [26-29]. When dNTPs bind in the active site, the tetramer is formed, and the tetramer was suggested to be long-lived in the cell [30]. SAMHD1 tetramer could maintain the cellular dNTP concentrations at a very low level outside the S phase of the cell cycle. Second, SAMHD1 was shown to have nuclease activity. The nuclease activity was localized to the HD domain of SAMHD1 [31]. Both single-stranded DNA and RNA nuclease activities have been reported for SAMHD1 [29,32].

Regulation of SAMHD1 occurs by three mechanisms. First, promoter methylation was shown to inhibit transcription [33], leading to a reduction in SMAHD1 levels. Second, SAMHD1 is regulated during S phase [34,35], being targeted for degradation. Third, SAMHD1 phosphorylation at T592 [35] was shown to regulate its antiviral activity but not dNTP phosphohydrolase activity [11]. Importantly, White *et al.* have shown non-dividing cells do not phosphorylate SAMHD1 at T592, whereas cycling cells do [11].

HIV-2 and some SIV strains encode for the accessory viral protein X (Vpx). It has the ability to target human SAMHD1 to the proteasome for degradation by DCAF1-E3-ubiquitin ligase [36-38]. Vpx interacts with the C-terminus of SAMHD1 in order to facilitate this degradation [14,39,40]. Recent reports have examined the acute kinetics of Vpx-mediated SAMHD1 degradation in myeloid cells and the enhancement of HIV-1 infection after Vpx treatment [8,41]. Further, we have reported in detail the acute effects of Vpx-mediated SAMHD1 degradation in monocyte-derived macrophages (MDMs) [42], which led to increased dNTP levels followed by enhancement of proviral DNA synthesis and transduction of MDMs.

In this report we performed an extensive kinetic and quantitative analysis examining the prolonged changes in dNTP concentrations and SAMHD1 levels over 14 days in primary human MDMs. In addition, we treated macrophages either with a single or dual VLP treatments, i.e. after the single Vpx + VLP treatment that kept SAMHD1 levels reduced by Western blots, and then measured dNTP levels and cellular nucleotide metabolites. While SAMHD1 remained very low, a second Vpx + VLP treatment promoted a rapid and robust increase in dNTPs. Collectively these data suggest that a very low level of SAMHD1 can dramatically modulate the dNTP concentrations in primary human MDMs.

## Results

### Monitoring the long-term kinetics of SAMHD1 levels and dNTP concentrations in human primary monocyte-derived macrophages

**Figure 1:**
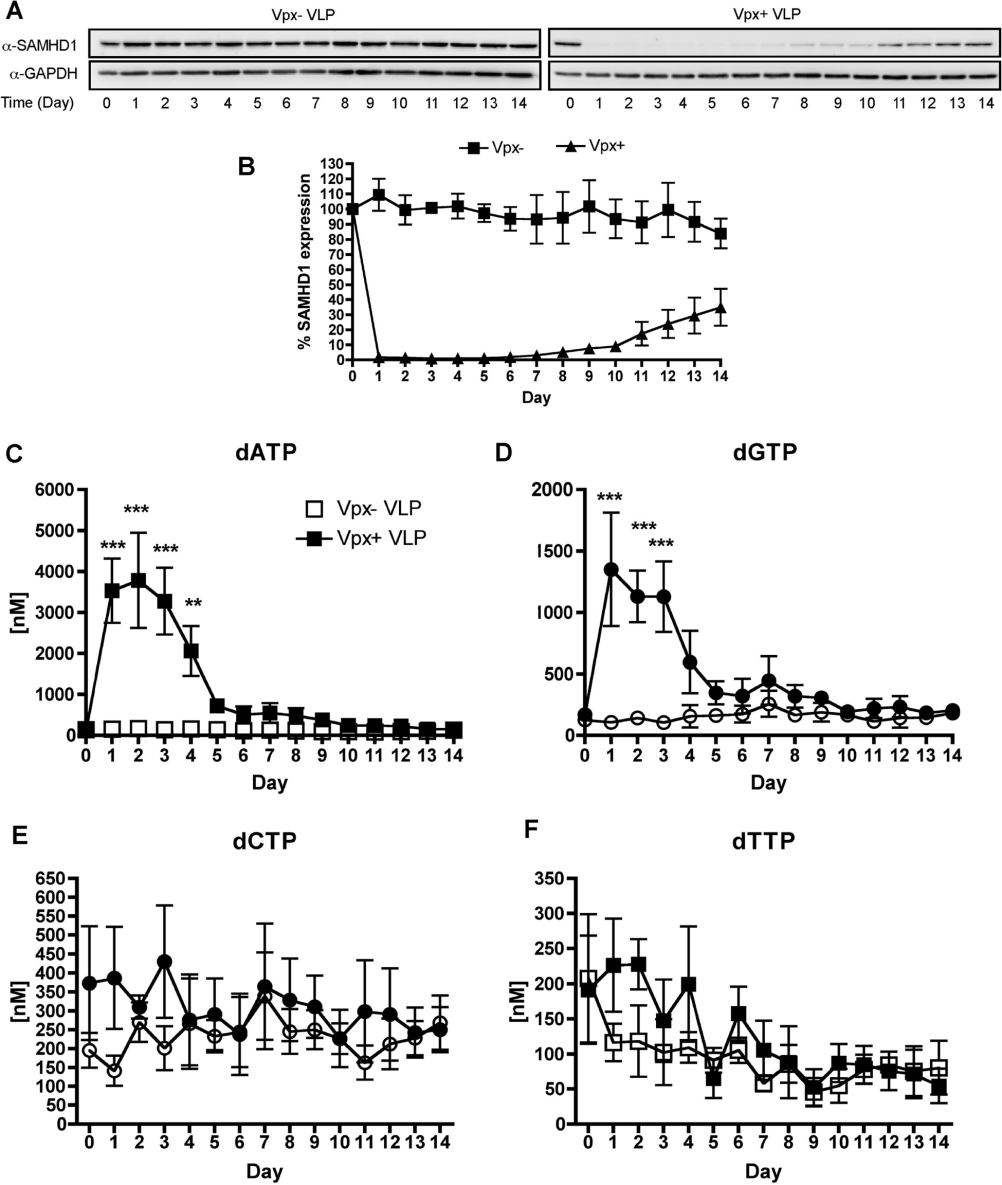
**Analysis of single VLP treatment in MDMs. (A)** MDMs were treated with Vpx- or Vpx + VLPs starting at day 0 and cell lysates were collected every 24 h until day 14. Immunoblots were probed for SAMHD1 or GAPDH, loading control. One representative MDM donor of three is displayed. **(B)** Quantitation of SAMHD1 expression (20 μg) by Western blot analysis is shown for the three independent MDM donors. Data are graphed by setting SAMHD1 expression level at day 0 to 100%. Two-way ANOVA was done with Bonferroni post-test analysis to determine significance, *** = P < 0.001; ** = P < 0.01. **(C)** Western blot analysis using 60 μg total protein, 3× loading as compared to **(B)**. SAMHD1 level of expression is below 3% even with greater loading. **(D-G)** HIV RT-based dNTP assay was used to determine the levels of dATP **(D)**, dGTP **(E)**, dCTP **(F)** and dTTP **(G)**. Data are graphed for the 15 days that the samples were collected. Two-way ANOVA analysis was performed and significant differences denoted on the graphs for dATP and dGTP.

We previously reported the acute effects of Vpx containing virus-like particles (VLP) treatment on human primary MDMs out to 48 h [42] and observed that the dGTP levels had peaked at day 1 and already started to decline at day 2 post Vpx treatment, while the SAMHD1 protein remained undetectable by western blot analysis. Many investigators have reported the effects of brief Vpx exposures in various cell types [8,18,19,41-43]. In this study, we investigated the recovery of SAMHD1 and changes in all four dNTP concentrations over 14 days in order to better understand cellular biology of SAMHD1 in primary human MDM treated with Vpx- or Vpx + VLP. As shown in Figure 1A, SAMHD1 remained undetectable under the immunoblot conditions described in previous studies until day 5, which is consistent with the long half-life of Vpx [6]. Vpx has a 30 h half-life, suggesting it would be decayed by day 5 after VLP + VLP treatment. Quantitative analysis for the SAMHD1 protein level (20 μg) from three donors (Figure 1B) revealed that at day 6, the SAMHD1 level was less than 3% of the normal endogenous SAMHD1 level found in MDMs, which was confirmed by loading 3× (60 μg) the amount of protein at day 6 (Figure 1C). Importantly, SAMHD1 began to recover to a consistently detectable level around day 7 and continued to recover to only 30% expression at day 14 (Figure 1A). These data indicate that a single treatment of Vpx + VLP can induce a very prolonged phase of SAMHD1 reduction in MDMs followed by a slow recovery of protein.

Next, we employing our highly sensitive HIV-1 RT-based dNTP assay [44] to examine the effects on dNTP pools after Vpx-mediated SAMHD1 degradation in the same MDM donors used in Figure 1B. As shown in Figures 1D-G, increases in all four dNTPs were clearly detected after Vpx + VLP treatment in MDMs. dATP and dGTP concentrations (Figures 1D and 1E) were significantly increased, while dCTP and dTTP concentrations (Figures 1F and 1G) were only modestly increased. dGTP concentration peaked at day 1, consistent with our published results [42]. Importantly, dATP peaked at day 2 before the contraction began, indicating dNTP modulation was different for each of the nucleotides. The dNTP contraction occurred even though SAMHD1 protein remained undetectable at days 3–5 by Western blot analysis. Basically, the rapid dNTP retraction following its acute elevation by Vpx occurred much ahead of the SAMHD1 recovery. These data suggest that the decrease in dNTP levels may be independent of the total SAMHD1 protein level due to various possibilities, which are discussed below.

### Effect of dual Vpx + VLP treatment on dNTP levels in MDMs

**Figure 2:**
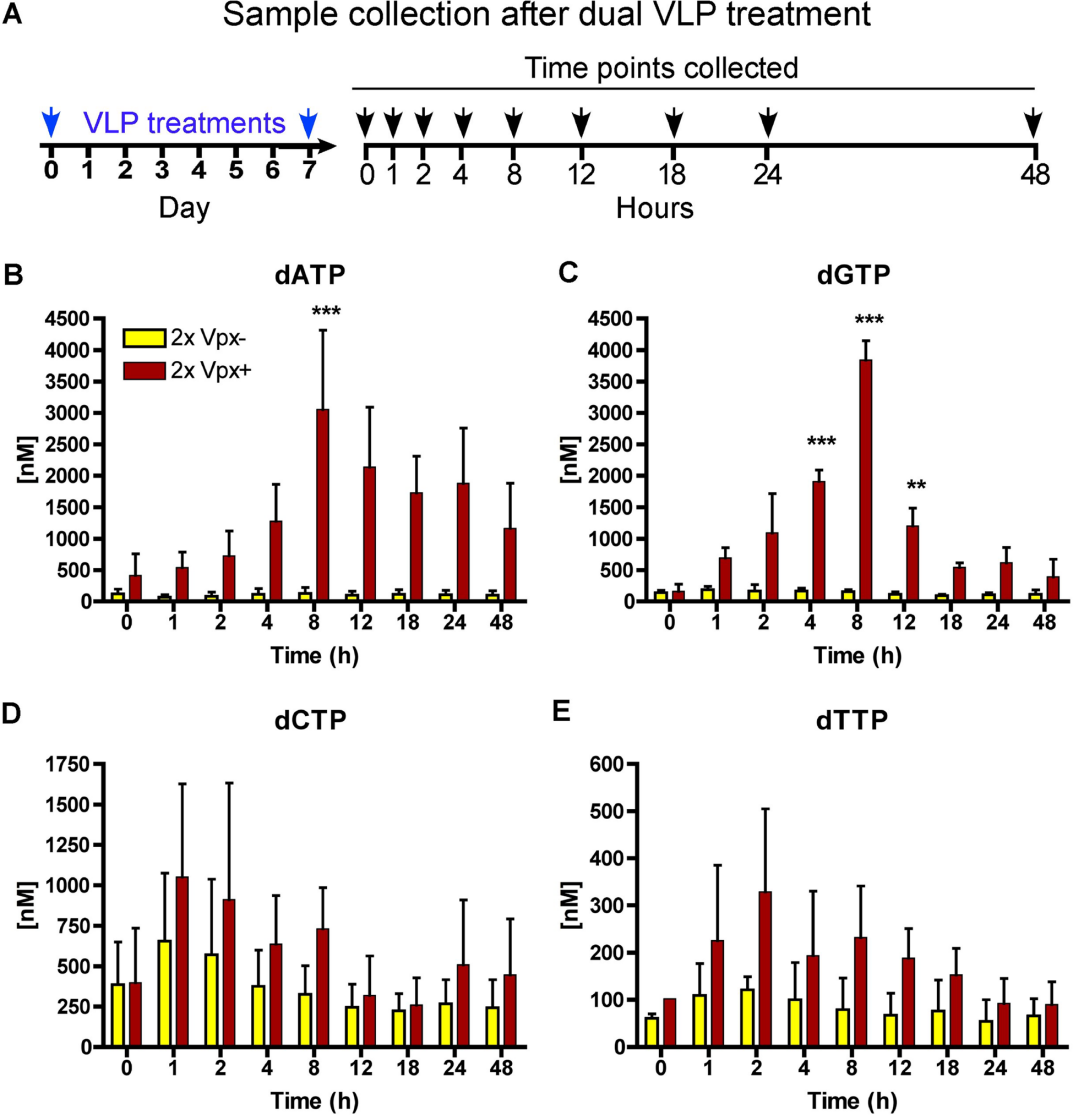
**Faster rise in dNTPs after dual Vpx + VLP treatment in MDMs. (A)** Diagram illustrating the experimental design. MDMs were treated with VLP on days 0 and 7, which were followed by cell collection for dNTPs over the next 48 h. **(B-E)** Analysis of dNTP concentrations using the RT-based assay. Significant differences in dATP **(B)** and dGTP **(C)** concentrations are shown when comparing the 2× Vpx- VLPs (yellow bars) and 2× Vpx + VLP (red bars) groups at 0, 1, 2, 4, 8, 12, 24, and 48 h post VLP addition. dATP **(A)** and dGTP **(B)** show a peak at 8 h after VLP addition, while dCTP **(C)** and dTTP **(D)** appeared to peak 1–2 h after addition. Significant differences were determined first using one-way ANOVA, followed by comparing each group using Mann–Whitney analysis. ***denotes P < 0.001.

The day 7 time point after single Vpx + VLP treatment of MDMs (Figure 1B) provided us with the unique opportunity to test whether dual (2×) Vpx + VLP treatment also influences the dNTP concentrations when SAMHD1 was less than 5% of the normal SAMHD1 level. For this test, dual Vpx + VLP treatment was performed at day 7 post primary VLP treatment and samples were collected every 24 h from days 7–14 (Additional file 1). We speculated that the peak in dNTP production during the dual Vpx + VLP treatment could have been missed while conducting kinetics analysis at 24 h time points. Therefore, as depicted in Figure 2A, we performed tighter acute kinetic dNTP analysis starting at day 7 after the dual Vpx + VLP treatment in the MDMs and looking within hours after dual treatment. As shown in Figures 2B-E, the second Vpx + VLP treatment showed an accelerated dNTP enhancement that peaked at around 8 h for dATP (Figure 2B) and dGTP (Figure 2C). Even more surprisingly, dCTP (Figure 2D) and dTTP (Figure 2E) peaked at 1–2 h post VLP treatment. Basically, the dual Vpx + VLP treated MDMs produced an accelerated increase in dNTP concentrations as compared to the single Vpx + VLP treatment [42]. Several possible scenarios may explain the faster dNTP elevation induced by the second Vpx + VLP treatment. First, in addition to its ability to promote SAMHD1 degradation, we speculate that Vpx may also activate the overall dNTP biosynthesis pathways. Alternatively, ribonucleotide reductase (RNR) activity through feedback modulation by cellular dATP was somehow changed from the primary and second Vpx + VLP treatment. This would help account for the fairly rapid contraction in dNTP concentrations after the second Vpx + VLP treatment. We did not see any modulation in the subunits of RNR after Vpx + VLP treatment (Additional file 2), but we have not been able to rule out Vpx potential modulation of RNR activity by an unknown mechanism. Another potential mechanism is that Vpx may target a putative minor SAMHD1 population that may be phosphorylated. Preliminary data suggest that MDMs can have a pSAMHD1 at T592 population, but both time in culture and SAMHD1 protein recovery after Vpx + VLP treatment showed the loss and the lack of phosphorylated SAMHD1, respectively (Additional file 3). This rules out modulation of dNTP concentrations by SAMHD1 phosphorylation during the recovery phase of SAMHD1 (days 7–14).

### Comparison of the effects of the single and dual Vpx + VLP treatments on HIV-1 transduction efficiency in MDMs

**Figure 3:**
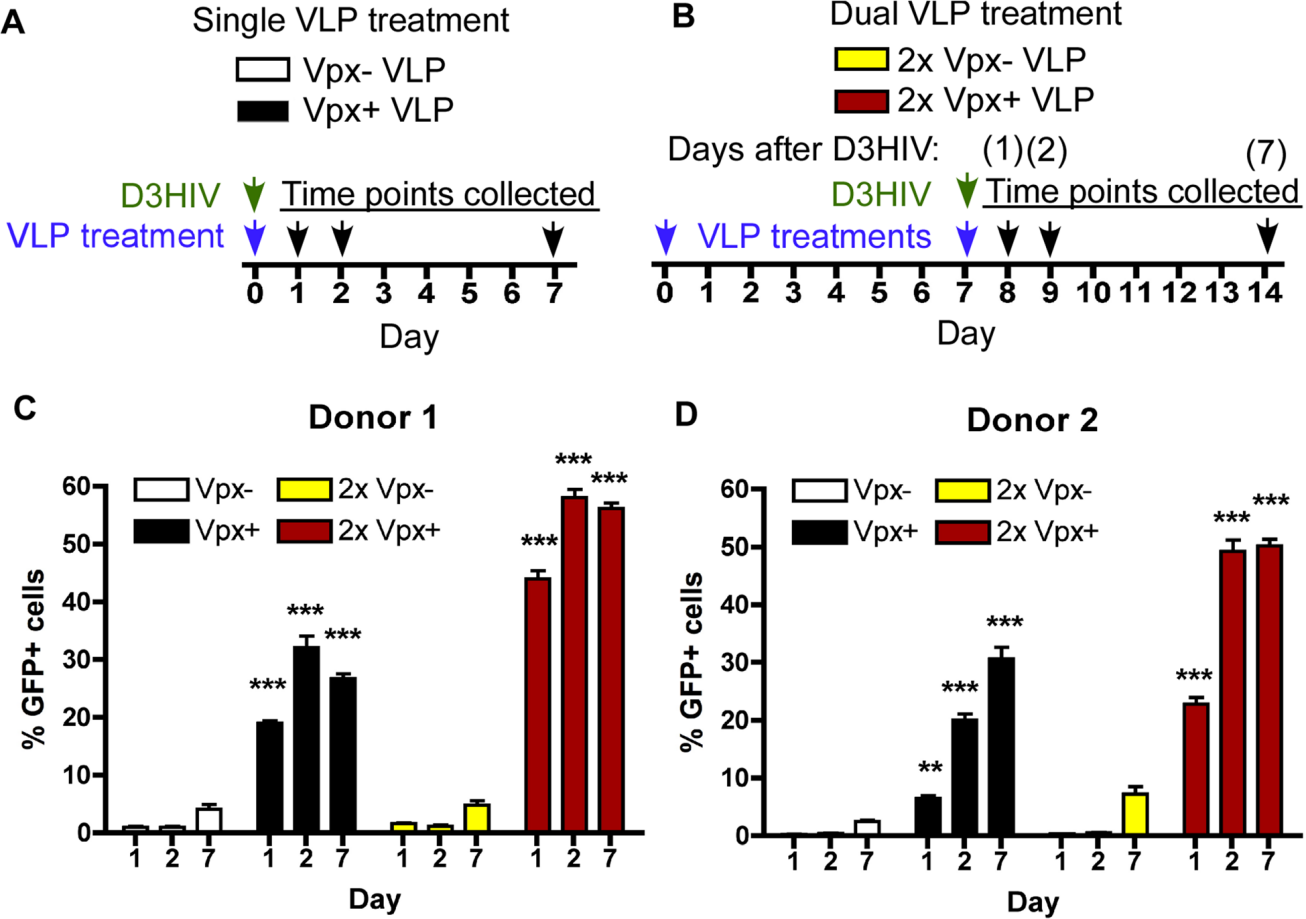
**Faster transduction in dual Vpx + VLP treated MDMs. (A)** Diagram showing the experimental procedure for single VLP treatment of MDMs. VLP and D3HIV treatments occurred at day 0, followed by collecting the cells for FACS analysis at days 1, 2 and 7. **(B)** An illustration depicting the experimental design with the dual VLP treatments occurring at days 0 and 7. D3HIV treatment was done at day 7. Cells were collected at days 8, 9 and 14, corresponding to days 1, 2 and 7, respectively, after D3HIV vector addition to the medium. **(C-D)** FACS analysis monitoring the frequency of GFP + MDMs for two independent donors treated with single VLP: Vpx- (white bars), Vpx + (black bars) or dual VLP: Vpx- (yellow bars) and Vpx + (red bars). Significant differences were determined first using one-way ANOVA, followed by comparing each group using Mann–Whitney analysis. ***denotes P < 0.001.

Next, we investigated how single and dual Vpx + VLP treatments affects HIV-1 transduction in MDMs. For this, we employed D3HIV-GFP vector that encodes the entire HIV-1 genome except for *env* and *nef,* which are deleted and replaced with *eGFP*. As shown in Figure 3A, single VLP treatment and D3HIV-GFP vector was added to the medium at day 0, followed by collection of the MDMs at days 1, 2 and 7 for FACS analysis. As illustrated in Figure 3B, MDMs were treated at days 0 and 7 with VLPs, and then D3HIV-GFP vector was added at day 7. MDMs were collected at days 8, 9 and 14 (corresponding to days 1, 2 and 7 after D3HIV-GFP vector addition). The vector transduction efficiency was measured by monitoring eGFP expression by FACS analysis from two independent MDM donors and graphed at days 1, 2 and 7 post D3HIV-GFP vector transduction (Figures 3C and 3D). MDMs treated with Vpx- VLP and D3HIV-GFP had about 5% transduction at day 7 post transduction (see white bars in Figure 3C and 3D). The single Vpx + VLP treatment (black bars in Figure 3C and 3D) enhanced HIV-1 vector transduction to ~30% transduction at day 7 as compared to the Vpx- VLP treatment (~5% at day 7). However, the dual Vpx + VLP treatment (red bars) was able to induce 50-60% transduction at day 7 as compared to the single Vpx + VLP treatment (black bars). Collectively, these data support that the faster dNTP increase by the dual Vpx + VLP treatment was able to further enhance HIV-1 transduction in MDMs.

### Measurement of dNTP intermediate metabolites by quantitative LC-MS/MS

**Figure 4:**
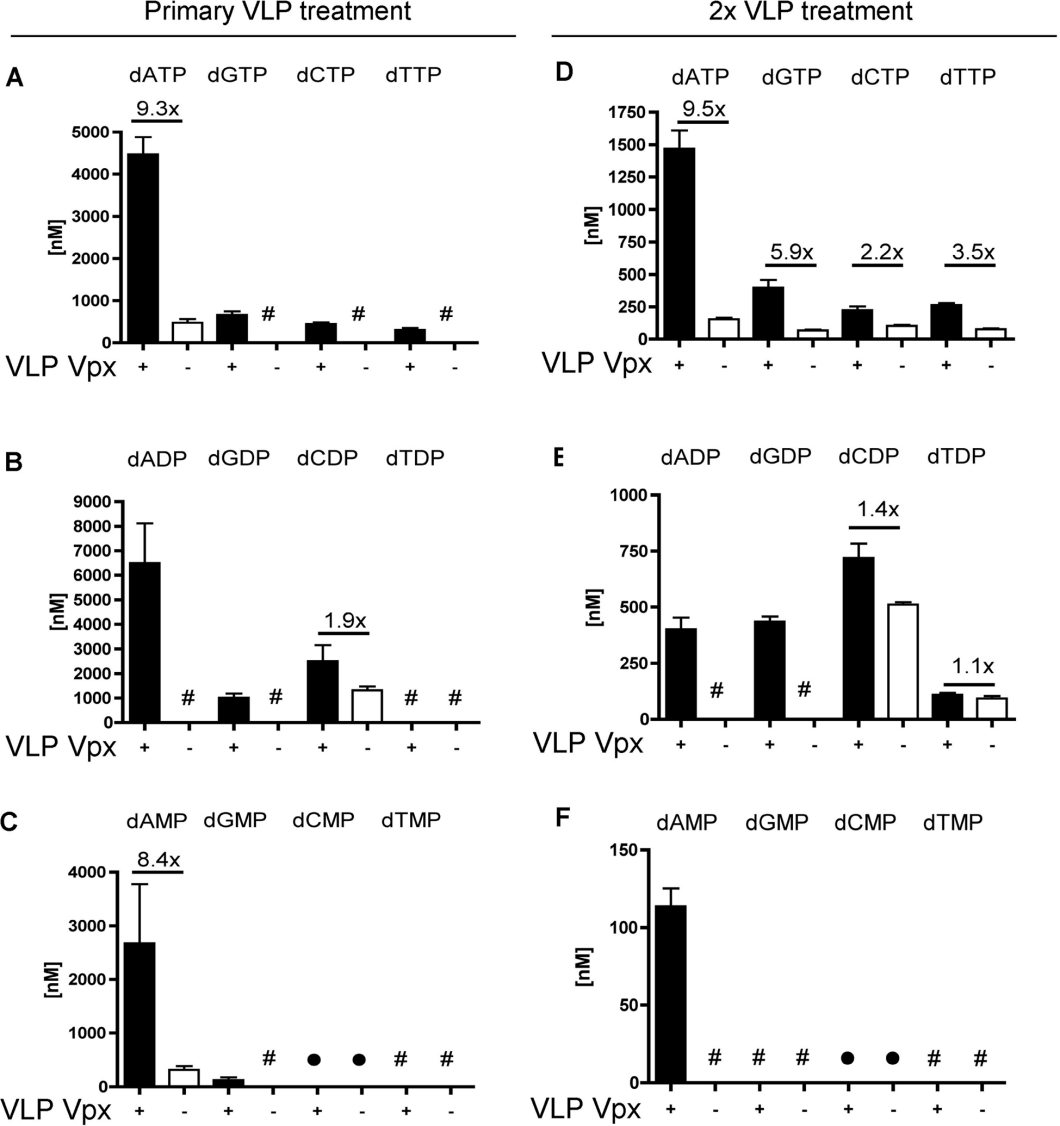
**LC-MS/MS analysis of single and dual VLP treated MDMs.** MDM donors were treated with a single or dual VLP. Cellular lysates were analyzed by LC-MS/MS analysis. Fold changes in deoxynucleoside metabolites **(A)** dNTPs, **(B)** dNDP, and **(C)** dNMP, are plotted for the single VLP treatment for two MDM donors. # denotes metabolites below the level of detection for LC-MS/MS analysis. Filled circles indicate metabolites that could not be accurately determined due to overlaying peaks with another unidentified metabolite. Three MDM donors treated with dual VLP have fold changes plotted **(D)** dNTP, **(E)** dNDP, and **(F)** dNMP levels.

The overall dNTP biosynthesis pathways has more intermediates than what our HIV RT-based dNTP assay can measure. Since we observed a faster dNTP elevation by the dual Vpx + VLP treatment as compared to the single Vpx + VLP treatment (Figure 2), and that the rapid dNTP retraction observed before any detectable SAMHD1 reappears (Figure 1), we monitored the metabolic changes in all three dNTP intermediate precursors: deoxynucleoside monophosphates (dNMPs) and diphosphates (dNDPs) and triphosphates (dNTPs) metabolites, by employing quantitative LC-MS/MS technology [45]. For these studies MDMs were treated with either single VLP doses and cells were harvested for dNTPs 24 h later. For the dNTP levels (Figure 4A), it is clear that the single Vpx + VLP treatment enhances the levels of and at least dADP, dGDP and dCDP, while dTDP was below the level of detection (see # in Figure 4B). But the dual Vpx + VLP treatment (Figure 4D-F) also induced greater dNDP levels, particularly purine dNDP as compared to the same MDM donor treated with Vpx- VLP. For dNMPs levels, only the dAMP measurement generated significantly detectable signals in both single Vpx + VLP (Figure 4C) and dual Vpx + VLP (Figure 4F) treatments in MDMs. Importantly, the LC-MS/MS analysis supports that Vpx + VLP treatment not only elevates the dNTP levels in MDMs but also the dNTP intermediate precursors. Since the medium lacks deoxyribonucleosides, we postulate via indirect evidence that the increase in dNDP metabolites has to occur through the activation of RNR. The exact mechanism remains unclear and is not linked to an increase in RNR subunit protein levels (Additional file 2). Moreover, Vpx is localized to the nucleus [5,10,14-19], suggesting that it does not have a direct interaction with RNR.

### Effect of gemcitabine, an RNR inhibitor, on the Vpx-mediated dNTP elevation in MDMs

**Figure 5:**
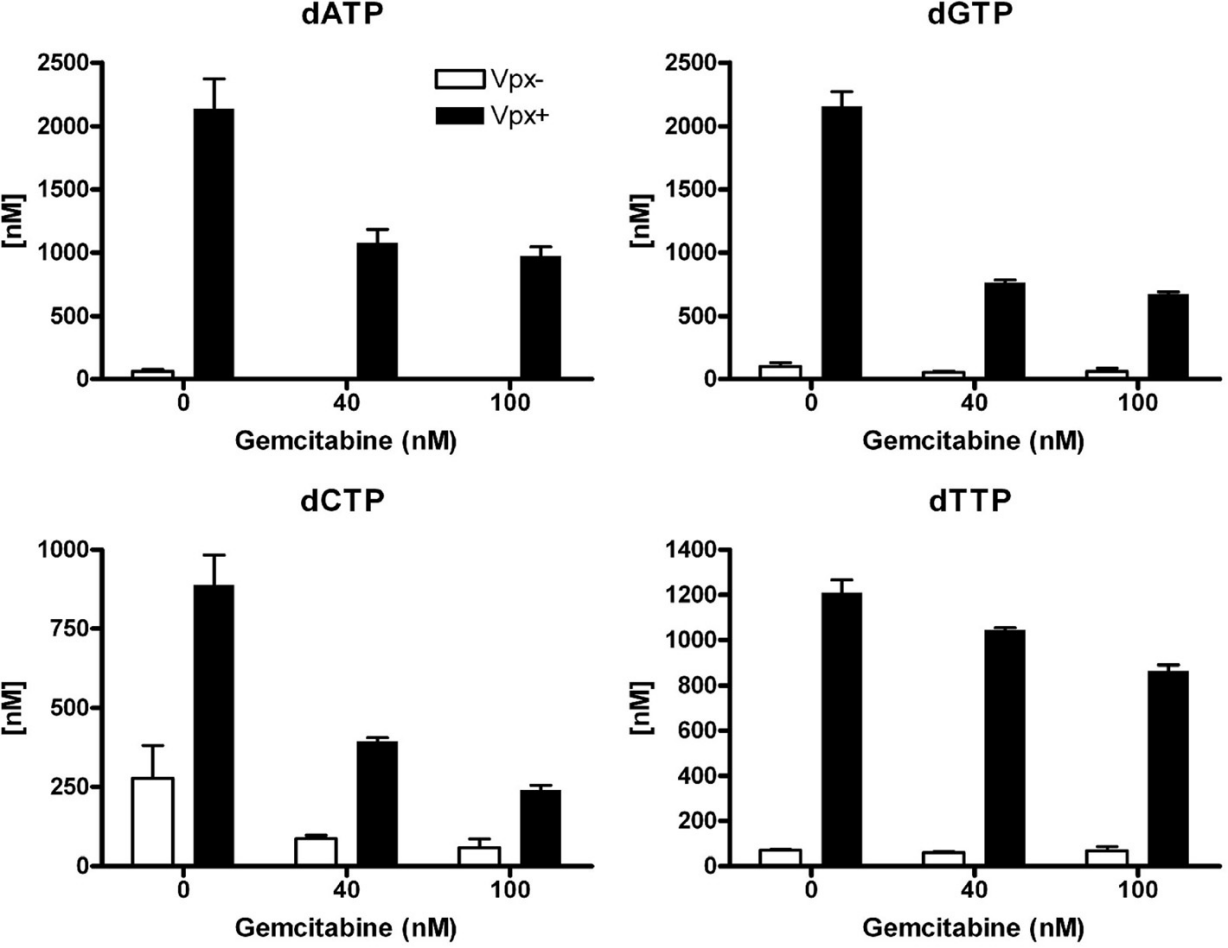
**Acute exposure of gemcitabine inhibits the further raise of dNTPs in single Vpx + VLP MDMs.** To further validate that gemcitabine does not have off target effects, MDMs were first treated with VLPs for 22.5 h before the addition of 40 and 100 nM gemcitabine to the medium. Samples were processed for dNTPs 1.5 h after gemcitabine addition. **(A)** dATP, **(B)** dGTP, **(C)** dCTP, and **(D)** dTTP concentrations were determined and means for two independent MDM donors plotted. Two-way ANOVA analysis was performed and significant differences denoted on the graphs. Importantly, acute exposure of gemcitabine for 1.5 h caused a much greater inhibition in the further raise of dCTP, dGTP and dATP levels treatment as compared to no gemcitabine control sample, while only having a modest influence on dTTP pool size.

It was previously demonstrated that hydroxyurea, an RNR inhibitor, could block Vpx-mediated increase in dNTPs [41]. We examined the contribution of RNR to increase the dNTP concentrations after Vpx-mediated SAMHD1 degradation in MDMs. We first tested dose-escalating concentrations of gemcitabine, a clinically available RNR inhibitor [46], for 24 h (Additional file 4). From these data, we tested 40 and 100 nM dose-escalating concentrations of gemcitabine. MDMs were pretreated 22.5 h with VLPs before adding gemcitabine. Cells were collected 1.5 h later and analyzed for changes in dNTP levels (Figure 5). We observe a 50% reduction in the levels of dATP (Figure 5A), dGTP (Figure 5B) and dCTP (Figure 5C) after 100 nM gemcitabine treatments, whereas dTTP concentration (Figure 5D) was less influenced. Collectively, these data indicate that RNR greatly contributes to the overall increase in Vpx-mediated dNTPs increase in the absence of SAMHD1.

## Discussion

This investigation began by examining how quickly SAMHD1 protein levels return after a single Vpx + VLP treatment in seven day maturated, primary human MDMs. We found that the level of SAMHD1 remained very low (less than 3% of the normal level in macrophages) between days 1–6 before it became consistently detectable by Western blot analysis at day 7. Vpx has been shown to have a long cellular half-life [6] and should be degraded roughly by day 5 after VLP treatment. However, SAMHD1 never recovered to its normal high level even at day 14, suggesting *de novo* protein synthesis of SAMHD1 may also be very slow or negatively regulated after Vpx treatment. Examining dNTP levels over this long time course showed a different observation. The levels of dNTPs were reduced well before the detection of SAMHD1 level by Western blot (Figure 1A), yet dNTPs declined starting at day 2 for dGTP, dCTP and dTTP and day 3 for dATP post Vpx + VLP treatment. Thus we speculate that additional factors or very low levels of SAMHD1 may be regulating dNTP pool sizes. We expected that the reduced level of SAMHD1 at day 7 post Vpx + VLP treatment would provide a window of opportunity to investigate cellular dNTP metabolism during a second Vpx + VLP exposure. Indeed, the dual Vpx + VLP treatment of MDMs at day 7 was able to display the same robust dNTP elevation at the time point when SAMHD1 remained less than 3% of the normal level in MDMs (Figure 2). We speculate that since the kinetics of the dual treatment are faster and the HLPC-MS data indicated an increase in dNDP metabolites, Vpx may harbor a SAMHD1 independent function that actively facilitates cellular dNTP biosynthesis metabolism and elevates cellular dNTP levels in the presence of only 3% of the normal SAMHD1 level. This potential mechanism is not related to the up regulations of RNR subunits (Additional file 2). Moreover, we have no direct evidence that Vpx interacts with any of the RNR subunits. Thus, it remains unclear as to just how Vpx VLP treatment and dNDP increase might be occurring.

Interestingly, the deoxypurine triphosphate (dATP and dGTP) concentrations remained high for several days after the single VLP treatment, while deoxypyrimidines – dCTP and dTTP concentrations showed only a transient increase before returning to base line levels for the majority of the MDM donors tested in this study (Figures 1E-F). Our data are consistent with the recently generated SAMHD1 deficient mouse [5], which shows that dATP and dGTP concentrations were significantly increased in the SAMHD1 deficient mice as compared to wild type mice. Importantly, the dual Vpx + VLP treatment of MDMs was informative by showing that the peak of the dATP and dGTP occurred around 8 h post Vpx + VLP addition, which is much faster than our results published for dGTP on the acute kinetics by the single Vpx + VLP treatment [42]. Interestingly, we observed a decline in the levels of all dNTPs at day 3 post Vpx + VLP treatment, suggesting that turnover of the dNTP pool occurs by other underlying mechanisms other than SAMHD1. These other mechanisms may include hydrolysis by deoxynucleoside diphosphatases [47,48], shut-off of RNR activity, or conversion to energy currency for other cellular enzyme reactions. Since MDMs are non-dividing cells, we can rule out that the decrease in dNTPs after a dual Vpx + VLP treatment is due to the dNTP utilization during DNA replication. However, we cannot rule out that DNA repair activity is occurring and is consuming the dNTPs. We postulate that the elimination of SAMHD1 may lead to establishing a new modulation set point of dNTP pools within the cell, with dATP and dGTP concentrations remaining much higher than dCTP and dTTP levels [5].

## Conclusions

We employed a series of biochemical and virological investigations with extensive and multiple exposures of Vpx + VLP treatments to human primary MDMs. These studies revealed that there was a significant quantitative discord between levels of total SAMHD1 protein and cellular dNTPs when MDMs were treated with Vpx + VLP. One potential explanation is that Vpx may promote targeting of SAMHD1 for degradation but also facilitate dNTP biosynthesis in macrophages since we detect an increase in dNDP metabolites, which are the precursors for dNTPs. This in turn would achieve a rapid and robust dNTP elevation, which is necessary for accelerating lentiviral reverse transcription for HIV-2 and SIV and also DNA gap-filling repair as part of lentiviral integration [49], in cells having extremely low cellular dNTP abundance.

## Methods

### Ethics statement

These experiments used primary human monocytes obtained from human buffy coats (New York Blood Services, Long Island, NY). These are pre-existing materials that are publicly available, and there is no subject-identifying information associated with the material obtained from this supplier. As such, the use of these samples does not represent human subjects research because: 1) materials were not collected specifically for this study, and 2) we are not able to identify the subjects.

### Cells

Primary human monocytes were isolated from the peripheral blood buffy coats by positive selection using MACS CD14+ beads as previously described [50]. Monocytes were maturated into monocyte-derived macrophages (MDMs) in the presence of 5 ng/ml hGM-CSF (Miltenyl Biotec) treated at days 0 and 2 of maturation. MDMs were used at day 7 of maturation for experiments.

### Primer extension assay

Protocol was followed as previously described [42]. MDMs were lysed with 60% cold methanol. Cellular debris was cleared by 14 K rpm centrifugation. Supernatant was dried using a SpeedVac (Thermo Scientific). Pellets were resuspended in 20 μl water. Two microliters of sample were used in the primer extension assay. 5’ ^32^P-end-labeled primer (“P”; 5’-GTCCCTCTTCGGGCGCCA-3’) was individually annealed to one of four different templates (3‘-CAGGGAGAAGCCCGCGGTN-5’). The template:primer complex was extended by HIV-1 reverse transcriptase, generating one additional nucleotide extension product (“P + 1”) for one of four dNTPs contained in the dNTP samples extracted form the cells. In this assay, the molar amount of the P + 1 product is equal to that of each dNTP contained in the extracted samples, which allows us to calculate and compare the dNTP concentrations for the different treatments [44].

### VLP generation

T225 flasks containing 293FT cells were transfected with 40 μg of pVpx- VLP or pVpx + VLP (kindly provided by Drs. Florence Margottin-Goguet and Nathaniel Landau) and 20 μg of pVSVg at a ratio of 1 μg of DNA to 3 μl of polyethylenimine (1 mg/ml). The following day, medium was discarded and replaced with fresh DMEM medium (5% FBS and antibiotics). On days 2–3 after transfection, the medium was collected and replaced with fresh medium. On the day of collection, medium was centrifuged at 1200 rpm for 5 min to remove cells. Supernatant was subsequently filtered through a 0.45-μm membrane (Corning Inc.) and overlaid on top of 5 ml of a 25% sucrose cushion (25% (w/v) sucrose, 10 mM Tris–HCl [pH 7.5], 0.1 M NaCl and 1 mM EDTA). VLPs were concentrated at 28,000 rpm for 90 min by ultracentrifugation. Supernatant was aspirated, and pellets were resuspended in 600 μl of serum-free DMEM. Supernatant was centrifuged for 1 min at 14 K rpm to remove debris. Aliquots (50 μl) were stored at −80°C. The p27 antigen level was determined using an ELISA kit (Advanced BioScience Laboratories, Inc., Rockville MD). A minimum of 145 ng of p27/million cells was used in experiments.

### D3HIV-GFP generation

pD3HIV-GFP vector encodes the HIV-1 NL4-3 genome with the eGFP gene in place of the HIV-1 *nef* gene and has a deleted envelope gene [44]. To generate virus, 293FT cells in T225 flasks were transfected with 60 μg pD3HIV-GFP and 20 μg pVSV-g using 140 μl polyethyenimine (1 mg/ml) in 37 ml DMEM medium/flask. At day 1 of HIV-1 production, medium was discarded and replaced with fresh complete DMEM medium (5% FBS plus antibiotics). At day 2, the medium was harvested and replaced. The medium was centrifuged at 2500 rpm for 7 min to remove cellular debris, and then stored at 4°C in T75 flask. Day 3 medium was harvested and processed as described for day 2. D3HIV-GFP was concentrated using ultracentrifugation (22 K rpm for 2 h in a SW32 Ti rotor). Pellets were resuspended in 0.5 ml serum free DMEM medium. Afterwards, debris was removed by centrifugation (14 K for 2 min). Sample aliquots (50 μl) were frozen at −80°C until used. MDMs were transduced with D3HIV-GFP and then the samples were analyzed using Accuri C6 flow cytometer monitoring GFP expression at the indicated times. Data files were analyzed using FlowJo software (TreeStar).

### Mass spectrometry

MDM extracts were generated by scraping wells with 70% methanol and freezing them overnight at −80°C. Extracts were centrifuged at 13,000 × *g* for 3 min and the supernatants were subsequently dried. The resulting samples were reconstituted in HPLC mobile phase for LC-MS/MS analysis as described previously [51]. In short, samples were reconstituted in 200 μl of 2 mM NH_4_H_2_PO_4_ with 3 mM hexylamine then analyzed for deoxyribonucleosides. Samples were separated using Hypersil Gold 100 × 1 mm column using Mobile phase - A: acetonitrile and B: 2 mM NH_4_H_2_PO_4_ with 3 mM hexylamine. A increased from 5% to 50% in 10 min, keep 50% for 3 min. The *m*/*z* parent to product MS/MS transitions: 523 to 146, 539 to 162, 496 to 119, and 495 to 81 were applied for the standard stable labeled isotopes and 508 to 136, 524 to 152, 484 to 112, and 485 to 81 for the corresponding sample nucleotides, respectively.

Samples were reconstituted in 200 μl of 2 mM NH_4_H_2_PO_4_ with 3 mM hexylamine_,_ and then split into two fractions. One fraction was analysis for deoxyribonucleosides. Samples were separated using Hypersil Gold 100 × 1 mm column using Mobile phase - A: acetonitrile and B: 2 mM NH_4_H_2_PO_4_ with 3 mM hexylamine. A increased from 5% to 50% in 10 min, keep 50% for 3 min. Instrument parameters were optimized for each metabolite (Additional file 5).

### Western blot analysis

Samples were processed in RIPA buffer containing 1 μM DTT, 10 μM PMSF, 10 μl/ml phosphatase inhibitor (Sigma) and 10 μl/ml protease inhibitor (Sigma). The cells were sonicated with 3×, 5 second pulses, to ensure complete lysis. Cellular debris was removed by 15 K rpm centrifugation for 10 min. Supernatants were stored at −80°C before use. Cell lysates were resolved on a 8% SDS-PAGE gel. Proteins were transferred to nitrocellulose membrane and detected as described in the figure legends using the following antibodies: rabbit anti-SAMHD1 mAb (Abcam), anti-GAPDH mouse mAb (Santa Cruz). Anti-mouse and anti-rabbit secondary HRP antibodies were purchased from GE HealthScience. HRP was detected using chemiluminescent reagents (Pierce) following the manufacturers instructions. Images were captured using BioRad ChemiDoc Imager. Anti-pSAMHD1 T592 antibody was obtained from Dr. Diaz-Griffero.

### Graphing and statistical analysis

Prism software was used for plotting the data. All the data sets were compared for significant difference using two-way ANOVA analysis and Bonferroni post-test analysis for significance.

## Additional files

Additional file 1:
**Dual Vpx + VLP Kinetic Analysis.** (A) Diagram showing the treatment regime for single and dual VLP treatments and the days harvested after treatment. (B-E) The HIV-1 RT-based dNTP assay was performed on samples to determine concentrations of the four different metabolites. Two-way- ANOVA was applied to determine significant differences within each group indicated (*, P < 0.05; **, P < 0.01 and ***, P < 0.001). Analysis was done for two independent MDM donors. Additional file 2:
**Analysis of RNR subunits.** MDMs were treated with VLP for 24 h and then processed for cellular lysates. Western blot analysis was performed using 25 μg total protein to detect the major subunit, R1 (Abcam), or the small subunits R2 and p53R2 (Santa Cruz). No significant changes in RNR expression levels were detected. Additional file 3:
**Analysis of pSAMHD1 at 592.** Two independent MDM donors were examined for pSAMHD1 by immunoblot analysis (20 μg protein). Total SAMHD1 was determined and GAPDH was used as the internal loading control. Additional file 4:
**Gencitabine inhibition of Vpx-mediated dNTP increase.** MDMs were pretreated for 2 h with various concentration of gemcitabine as indicated in figures. Next, VLPs were added and cells were placed in the incubator for 24 h, after which time they were processed for dNTPs. The HIV-1 RT-based dNTP assay was done and data plotted for each metabolite: (A) dATP, (B) dGTP, (C) dCTP and (D) dTTP. One-way ANOVA was done and significant differences indicated (*, P < 0.05; **, P < 0.01 and ***, P < 0.001). Analysis was done for two independent donors. Additional file 5:
**HPLC-MS settings for instrument optimization.** Each metabolite was purchased from Sigma and then ran on the HLPC-MS to optimize the reading. Then setting were then used to generate a concentration curve that was applied to determine the concentrations of these metabolites in MDMs treated with Vpx + VLP and Vpx- VLPs.
