# Supplementary material for: dNTP pool modulation dynamics by SAMHD1 protein in monocyte-derived macrophages
Source: Retrovirology. 2014 Aug 27;11:63. doi: 10.1186/s12977-014-0063-2 (PMC4161909; doi:10.1186/s12977-014-0063-2)
Supplement: Additional file 1: — Dual Vpx + VLP Kinetic Analysis. (A) Diagram showing the treatment regime for single and dual VLP treatments and the days harvested after treatment. (B-E) The HIV-1 RT-based dNTP assay was performed on samples to determine concentrations of the four different metabolites. Two-way- ANOVA was applied to determine significant differences within each group indicated (*, P < 0.05; **, P < 0.01 and ***, P < 0.001). Analysis was done for two independent MDM donors. [file 12977_2014_63_MOESM1_ESM.pdf]

Hollenbaugh et al., dNTP Pool Modulation Dynamics by SAMHD1 Protein in Monocyte-derived Macrophages

Additional file 1

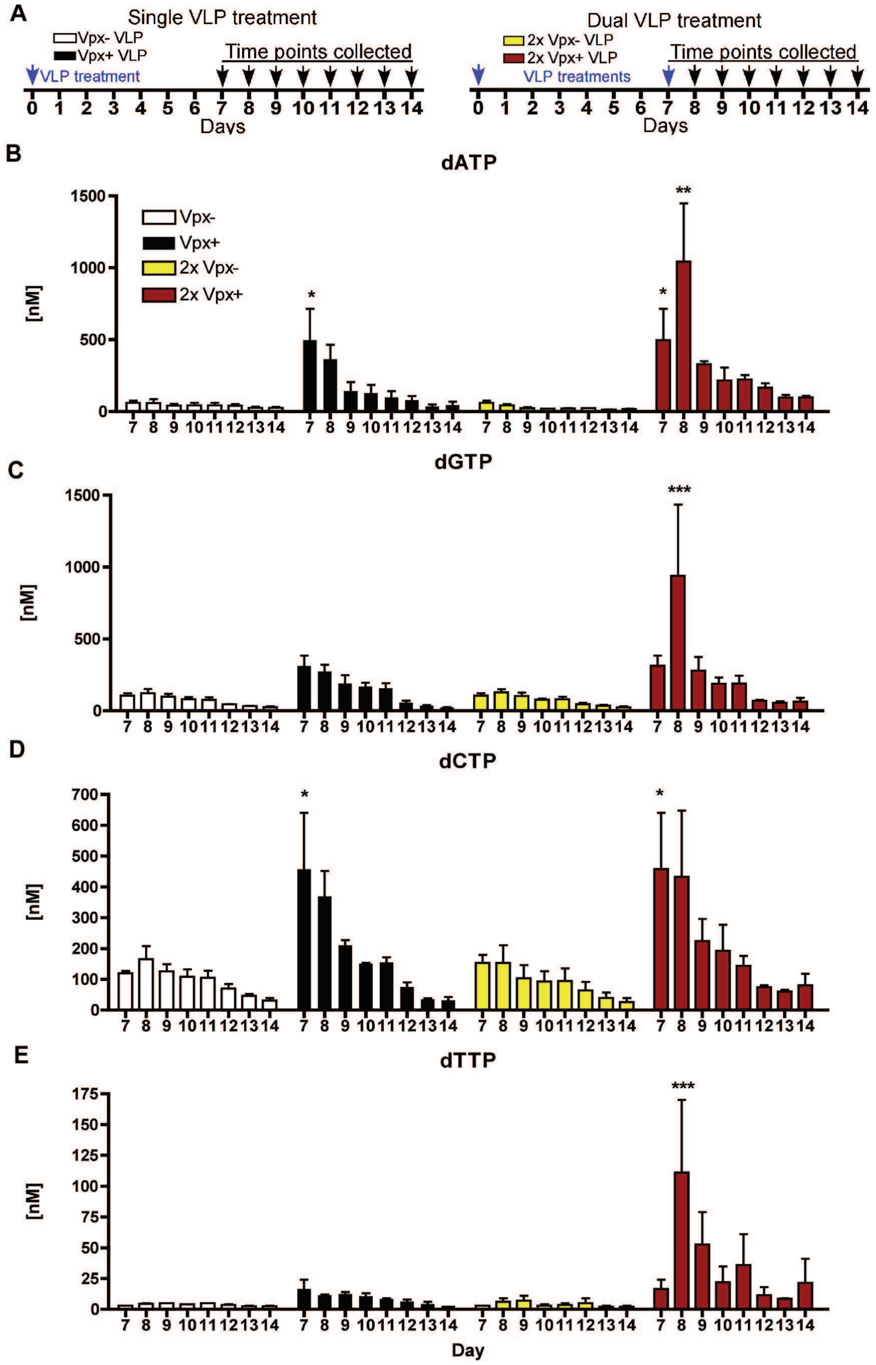

Additional file 1: Dual Vpx+ VLP Kinetic Analysis. (A) Diagram showing the treatment regime for single and dual VLP treatments and the days harvested after treatment. (B-E) The HIV-1 RT-based dNTP assay was performed on samples to determine concentrations of the four different metabolites. Two-way-ANOVA was applied to determine significant differences within each group indicated (*, P<0.05; **, P<0.01 and ***, P<0.001). Analysis was done for two independent MDM donors.
