# Supplementary material for: dNTP pool modulation dynamics by SAMHD1 protein in monocyte-derived macrophages
Source: Retrovirology. 2014 Aug 27;11:63. doi: 10.1186/s12977-014-0063-2 (PMC4161909; doi:10.1186/s12977-014-0063-2)
Supplement: Additional file 2: — Analysis of RNR subunits. MDMs were treated with VLP for 24 h and then processed for cellular lysates. Western blot analysis was performed using 25 μg total protein to detect the major subunit, R1 (Abcam), or the small subunits R2 and p53R2 (Santa Cruz). No significant changes in RNR expression levels were detected. [file 12977_2014_63_MOESM2_ESM.pdf]

Additional file 2

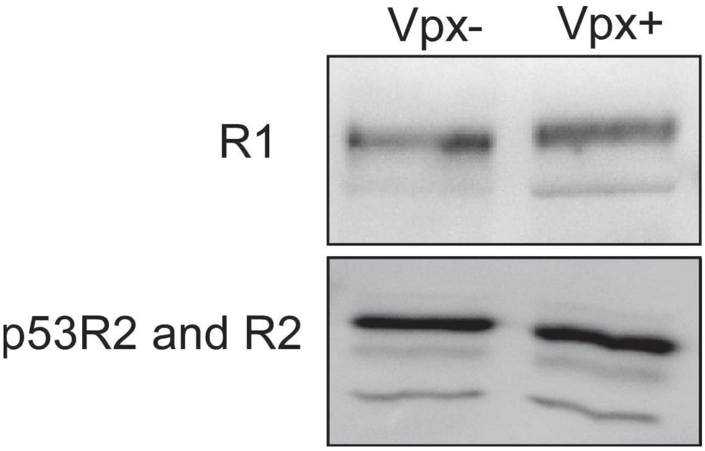

Additional file 2: Analysis of RNR subunits. MDMs were treated with VLP for 24 h and then processed for cellular lysates. Western blot analysis was performed using 25 µg total protein to detect the major subunit, R1 (Abcam), or the small subunits R2 and p53R2 (Santa Cruz). No significant changes in RNR expression levels were detected.
