# Supplementary material for: dNTP pool modulation dynamics by SAMHD1 protein in monocyte-derived macrophages
Source: Retrovirology. 2014 Aug 27;11:63. doi: 10.1186/s12977-014-0063-2 (PMC4161909; doi:10.1186/s12977-014-0063-2)
Supplement: Additional file 3: — Analysis of pSAMHD1 at 592. Two independent MDM donors were examined for pSAMHD1 by immunoblot analysis (20 μg protein). Total SAMHD1 was determined and GAPDH was used as the internal loading control. [file 12977_2014_63_MOESM3_ESM.pdf]

Hollenbaugh et al., dNTP Pool Modulation Dynamics by SAMHD1 Protein in Monocyte-derived Macrophages

Additional file 3

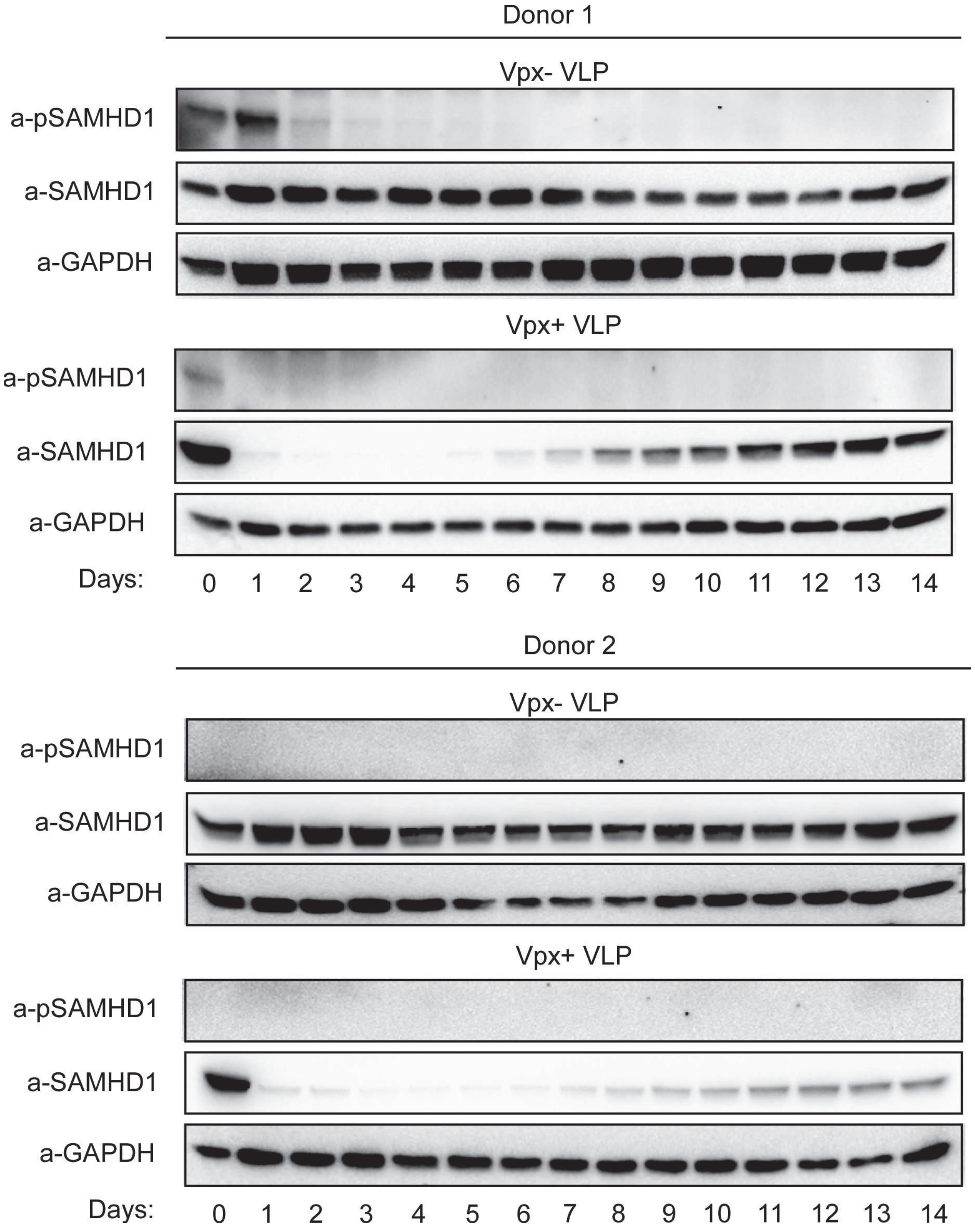

Additional file 3: Analysis of pSAMHD1 at 592. Two independent MDM donors were examined for pSAMHD1 by immunoblot analysis (20 µg protein). Total SAMHD1 was determined and GAPDH was used as the internal loading control.
