# Supplementary material for: dNTP pool modulation dynamics by SAMHD1 protein in monocyte-derived macrophages
Source: Retrovirology. 2014 Aug 27;11:63. doi: 10.1186/s12977-014-0063-2 (PMC4161909; doi:10.1186/s12977-014-0063-2)
Supplement: Additional file 4: — Gencitabine inhibition of Vpx-mediated dNTP increase. MDMs were pretreated for 2 h with various concentration of gemcitabine as indicated in figures. Next, VLPs were added and cells were placed in the incubator for 24 h, after which time they were processed for dNTPs. The HIV-1 RT-based dNTP assay was done and data plotted for each metabolite: (A) dATP, (B) dGTP, (C) dCTP and (D) dTTP. One-way ANOVA was done and significant differences indicated (*, P < 0.05; **, P < 0.01 and ***, P < 0.001). Analysis was done for two independent donors. [file 12977_2014_63_MOESM4_ESM.pdf]

Hollenbaugh et al., dNTP Pool Modulation Dynamics by SAMHD1 Protein in Monocyte-derived Macrophages

Additional file 4

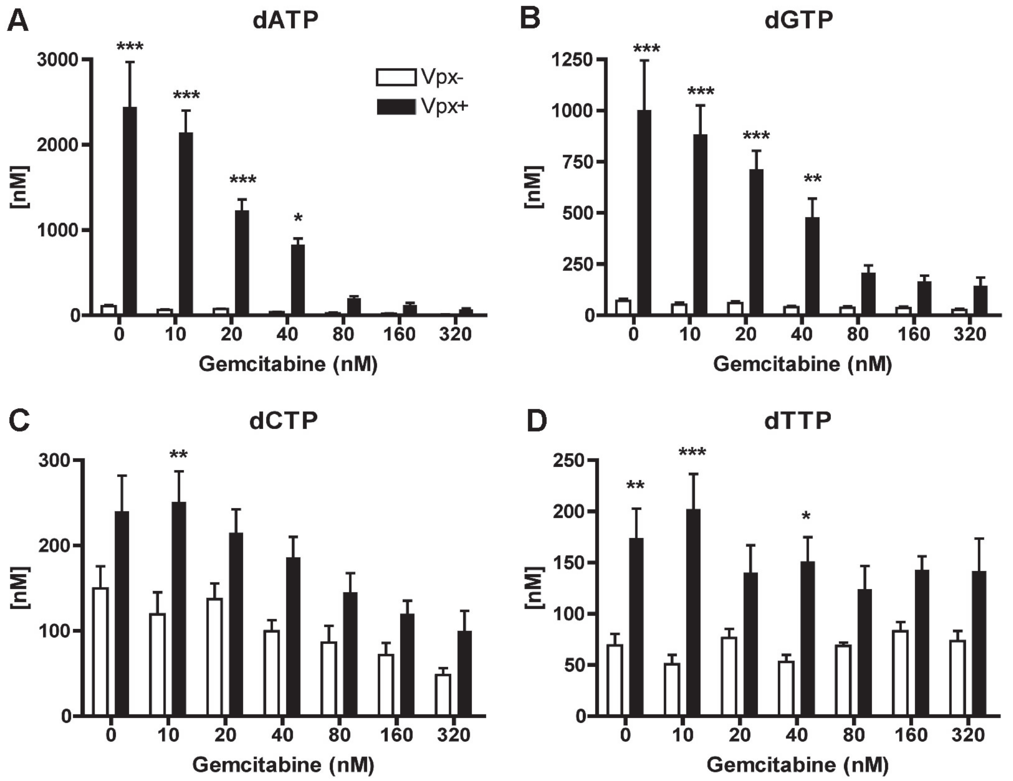

Additional file 4: Gencitabine inhibition of Vpx-mediated dNTP increase. MDMs were pretreated for 2 h with various concentration of gemcitabine as indicated in figures. Next, VLPs were added and cells were placed in the incubator for 24 h, after which time they were processed for dNTPs. The HIV-1 RT-based dNTP assay was done and data plotted for each metabolite: (A) dATP, (B) dGTP, (C) dCTP and (D) dTTP. One-way ANOVA was done and significant differences indicated (*, $P<0.05$; **, $P<0.01$ and ***, $P<0.001$). Analysis was done for two independent donors.
