# Supplementary material for: dNTP pool modulation dynamics by SAMHD1 protein in monocyte-derived macrophages
Source: Retrovirology. 2014 Aug 27;11:63. doi: 10.1186/s12977-014-0063-2 (PMC4161909; doi:10.1186/s12977-014-0063-2)
Supplement: Additional file 5: — HPLC-MS settings for instrument optimization. Each metabolite was purchased from Sigma and then ran on the HLPC-MS to optimize the reading. Then setting were then used to generate a concentration curve that was applied to determine the concentrations of these metabolites in MDMs treated with Vpx + VLP and Vpx- VLPs. [file 12977_2014_63_MOESM5_ESM.pdf]

Hollenbaugh et al., dNTP Pool Modulation Dynamics by SAMHD1 Protein in Monocyte-derived Macrophages

Additional file 5

| Analytes | Parent Ion | Product Ion | Collision Energy (v) | Tube Lens Offset (v) |
|---|---|---|---|---|
| dAMP + | 332.1 | 136 | 16 | 52 |
| dGMP + | 348.1 | 152 | 14 | 55 |
| dCMP + | 308 | 112 | 14 | 65 |
| TMP - | 321 | 79 | 50 | -90 |
| dADP + | 412 | 136 | 15 | 95 |
| dGDP + | 428 | 152 | 15 | 107 |
| dCDP + | 388 | 112 | 23 | 95 |
| TDP - | 401 | 158.9 | 27 | -81 |
| dATP + | 492 | 136 | 28 | 140 |
| dGTP + | 508 | 152 | 22 | 146 |
| dCTP + | 468 | 112 | 28 | 130 |
| TTP - | 481 | 158.9 | 30 | -89 |

**Additional file 5: HPLC-MS settings for instrument optimization.** Each metabolite was purchased from Sigma and then ran on the HLPC-MS to optimize the reading. Then setting were then used to generate a concentration curve that was applied to determine the concentrations of these metabolites in MDMs treated with Vpx+ VLP and Vpx- VLPs.
